# Loss of AIM2 expression promotes hepatocarcinoma progression through activation of mTOR-S6K1 pathway

**DOI:** 10.18632/oncotarget.9154

**Published:** 2016-05-04

**Authors:** Xiaomin Ma, Pengbo Guo, Yumin Qiu, Kun Mu, Lihui Zhu, Wei Zhao, Tao Li, Lihui Han

**Affiliations:** ^1^ Department of Immunology, Shandong University School of Medicine, Jinan 250012, China; ^2^ Department of Pathology, Shandong University School of Medicine, Jinan 250012, China; ^3^ Department of Gastroenterology, Provincial Hospital Affiliated with Shandong University, Jinan 250021, China

**Keywords:** absent in melanoma 2, inflammasome, hepatocellular carcinoma, mammalian target of rapamycin, tumor progression

## Abstract

Absent in melanoma (AIM2) is a member of the interferon-inducible HIN-200 protein family and is recently recognized to play an important dual role in both innate immunity and tumor pathology. However, the role of AIM2 in the development of hepatocellular carcinoma (HCC) remains to be clarified. Here we showed that AIM2 expression was significantly decreased in liver cancer tissues, and loss of its expression was significantly correlated with more advanced tumor progression. Exogenous overexpression of AIM2 in HCC cells suppressed mammalian target of rapamycin (mTOR)-S6K1 pathway and further inhibited proliferation, colony formation and invasion of HCC cells. On the contrary, block of AIM2 in HCC cells induced (mTOR)-S6K1 pathway activation and thus promoted HCC progression. Treatment with mTOR pathway inhibitor rapamycin further verified its contribution to HCC progression in AIM2 absent HCC cells. Thus, these data suggested that AIM2 played a critical role as a tumor suppressor and might serve as a potential therapeutic target for future development of AIM2-based gene therapy for human liver cancer. This study also paves a new avenue to treat AIM2-deficient cancer by suppression of mTOR.

## INTRODUCTION

Hepatocellular carcinoma (HCC) is the most frequent and aggressive primary tumor of the liver and has limited treatment options. Prognosis of this disease is poor because of its rapid progression and high metastatic rate. Surgical resection is considered as one of the standard curative therapy and provides a long-term survival of patients, but HCC still shows high postsurgical recurrence, rapid progression and extremely poor prognosis. It is in critical need of defining its molecular mechanism and identifying new therapeutic target for manipulation of this disease.

Absent in melanoma 2 (AIM2) is a pyrin-HIN protein that binds cytosolic double-stranded DNA (dsDNA), and associates with ASC and caspse-1 to induce the formation of a large multiprotein molecular platform named inflammasome. Once stimulated, caspase-1 is activated and subsequently cleaves the proinflammatory IL-1 family of cytokines, IL-1β and IL-18 into their bioactive forms, and further causes a type of inflammatory cell death called pyroptosis. Besides innate immune cells, activation of AIM2 inflammasome in response to cytoplasmic dsDNA was also shown in non-myeloid cells including keratinocytes, vascular endothelia and smooth muscle cells, which indicated its versatile biological activities [1–3].

Though the function of AIM2 in innate immunity is well accepted, its role in cancer is less clear. AIM2 was originally investigated in melanoma cells and its absence in melanoma tissues promoted disease progression [4]. Further studies also support the tumor suppressor role of AIM2 in several types of tumors, including colon cancer [5–8], breast cancer [9], and prostate cancer [10]. Thus it indicated that innate immune DNA sensor AIM2 was implicated as a potential tumor suppressor, whereas its mechanism is not fully understood. Recent work in colon cancer indicated that AIM2 restricted proliferation of colon cancer cells by reducing the activation of AKT pathway [11], which helps to understood its tumor suppressor function in colon cancer. However, the role of AIM2 in the development and progression of hepatocellular carcinoma has never been clarified.

In this study, we detected the expression of AIM2 in clinical liver cancer specimen, analyzed the correlation between its expression and disease progression, and further investigated its role in malignant behaviors of HCC cells in cellular and animal models. Our data surprisingly indicated that loss of AIM2 in HCC cells contributed to disease progression via mammalian target of rapamycin (mTOR)-S6K1 pathway activation, which may pave a new avenue to treat AIM2 deficient cancer by suppression of mTOR.

## RESULTS

### Expression of AIM2 in HCC tissues was significantly decreased compared with distal non-cancerous liver tissues

**Figure 1:**
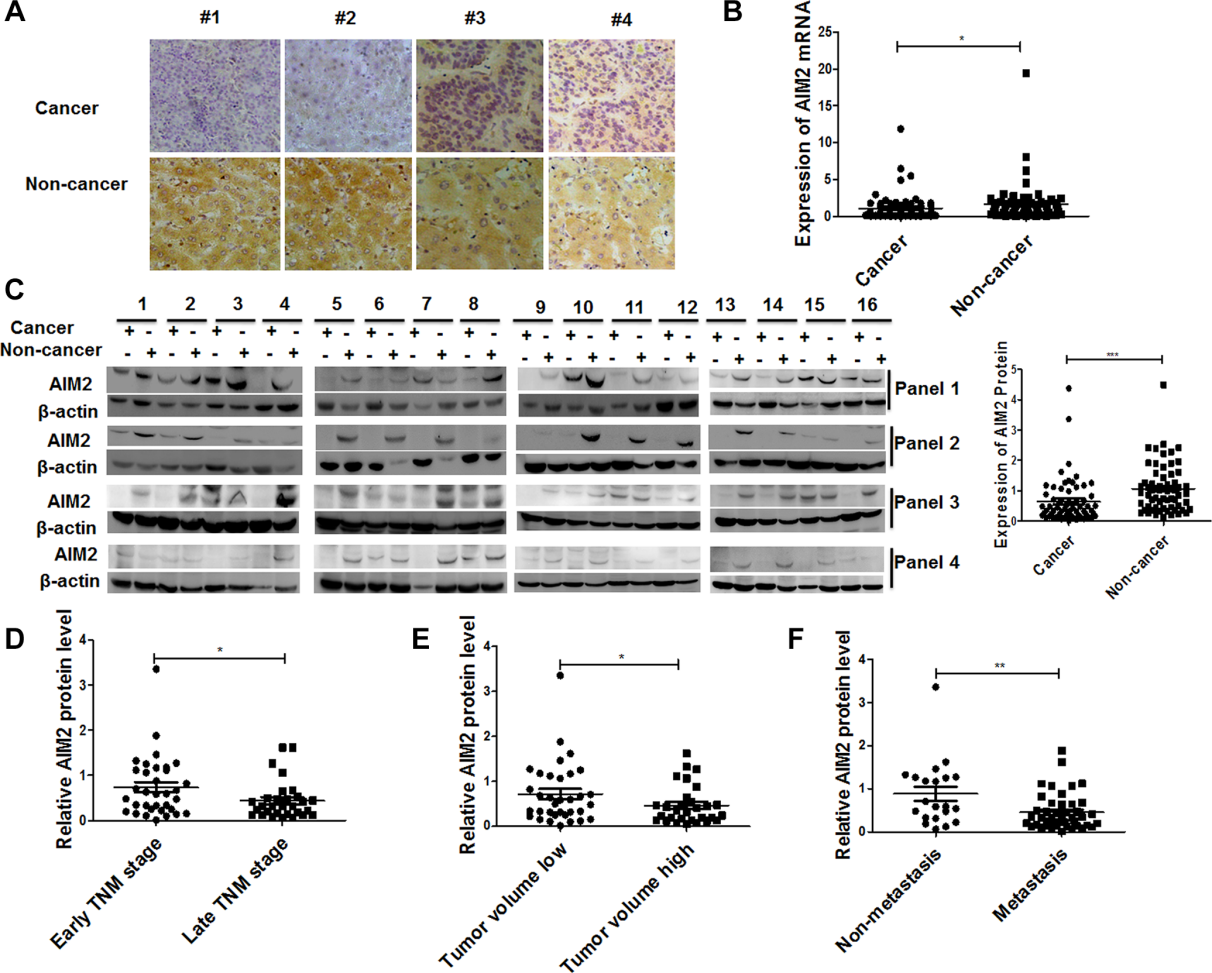
Expression of AIM2 protein and mRNA in HCC tissues and corresponding non-cancerous liver tissues (**A**) Immunohistochemical staining was used to determine the location and expression of AIM2 in HCC tissues and corresponding non-cancerous liver tissues. (**B**–**C**) AIM2 mRNA and protein expression in 64 pairs of cancerous and non-cancerous liver tissue samples was detected by Real-time PCR (B) and western blot (C), respectively. For the western blot assay, β-actin was used as a loading control. Statistical analysis of band intensity normalized to β-actin was shown in the right panel of Figure C. (**D**) The statistical analysis of AIM2 protein level in early TNM stage and late TNM stage HCC patients. (**E**) The relative AIM2 protein level was compared in small tumor group (the diameter < 5 cm) and larger tumor group (the diameter ≥ 5 cm). (**F**) Statistical analysis was performed to compare the AIM2 protein expression level in metastasis group and non-metastasis group. *P < 0.05, **P < 0.01, ***P < 0.001 for the statistical analysis of the indicated groups.

In order to define the expression status of AIM2 in liver cancer tissues, 113 clinical HCC patients were recruited for the investigation. Both of the excised liver cancer tissues and corresponding distal non-cancerous liver tissues were collected for further assay. Firstly, expression and location of AIM2 was detected by IHC in the liver cancer tissues and corresponding non-cancerous liver tissues from 49 HCC patients. Immunohistochemical staining showed that AIM2 signal was stained in yellow and brown, and it was mostly expressed in the cytoplasm of HCC cells and hepatocytes. Expression of AIM2 in the liver cancer cells were significantly decreased compared with corresponding distal non-cancerous liver tissues (Figure 1A, Table 1). Statistical analysis further confirmed that the expression level of AIM2 in HCC patients was significantly negatively correlated with tumor volume, Edmonson grade and TNM stages (Table 2). These data indicated that loss of AIM2 expression in liver cancer cells contributed to the disease progression of HCC patients.

**Table 1:** Expression of AIM2 in liver cancer tissues compared with non-cancerous liver tissues

	Cancer(%)	Non-cancer(%)	χ^2^	*P*-value
AIM2				
Low	87.8% (43/49)	20.4% (10/49)	44.747	0.0000
High	12.2% (6/49)	79.6% (39/49)		

Note: AIM2 Low = “–” “+”; High = “++” “+++”.

**Table 2:** Correlation analysis between the expression of AIM2 and clinical features

	Gender		Age(year)		Tumor diameter(cm)		Edmonson grade			TNM stage	
	Male	Female	< 54	≥ 54	< 5	≥ 5	I	II	III	Low	High
AIM2											
Low	38	5	20	23	15	28	3	30	10	20	23
High	6	0	2	4	6	0	4	2	0	6	0
	*r* = 0.126		*r* = 0.248		*r* = − 0.431		*r* = − 0.432			*r* = − 0.351	
	*p* = 0.389		*p* = 0.086		*p* = 0.02		*p* = 0.02			*p* = 0.013	

Note: AIM2 Low=“-”“+”High=“++”“+++” TNM Low=“I”“II”High=“III”“IV”.

The IHC data were further verified by western blot and qRT-PCR analysis in another cohort of 64 HCC patients with paired HCC tissues and corresponding distal non-cancerous liver tissues. These data verified that both of the protein level and mRNA level of AIM2 expression were significantly decreased in HCC tissues compared with corresponding distal non-cancerous liver tissues (Figure 1B–1C). Correlation analysis showed that protein levels of AIM2 were significantly positively correlated with mRNA levels (Supplementary Figure 1), which indicated the authenticity and consistency of these detection methods. Further analysis showed that loss of AIM2 expression in HCC tissues was significantly correlated with later TNM stages, larger tumor volumes and more advanced metastasis status (*P < 0.05, Figure 1D–1F). These data from clinical investigation altogether indicated that loss of AIM2 expression in HCC tissues may promote the disease progression of these patients.

### AIM2 inhibited proliferation, colony formation and invasion of HCC cells

We have shown that expression of AIM2 molecule was significantly decreased in HCC tissues compared with non-cancerous liver tissues, and then we tried to define whether loss of AIM2 expression contributed to HCC progression. So we investigated the effect of AIM2 on the malignant behaviors of these HCC cells.

Six different HCC cell lines were used to detect the relative expression of AIM2 (Supplementary Figure 2A–2B). Because SMCC7721 and HUH7 cells have relative lower levels of AIM2 expression, these two cell lines were transfected with AIM2 expression plasmid to investigate the effect of exogenous overexpression of AIM2 on HCC cells. On the other hand, because HepG2 and BEL7402 cells have relative higher levels of AIM2 expression, these two cell lines were transfected with siRNA specifically targeting AIM2 to investigate the effect of AIM2-knockdown on the HCC cells (Supplementary Figure 2C–2D). Three small interference RNAs were synthesized and after validation of their inhibitory efficiencies, two siRNA with inhibitory efficacy higher than 80% were selected and used for further study (Supplementary Figure 2E–2F).

**Figure 2:**
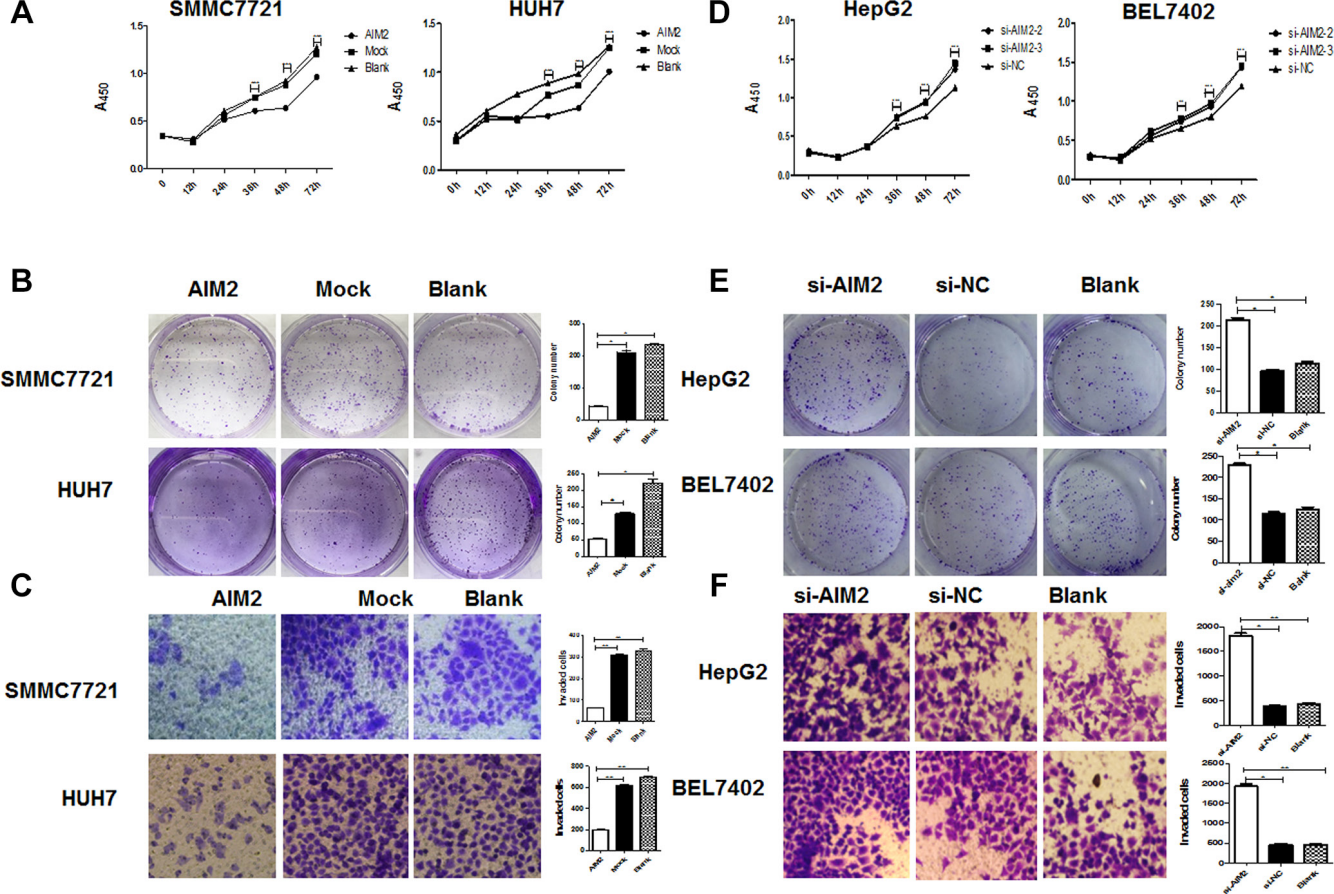
AIM2 suppressed the proliferation, colony formation and invasion of HCC cells (**A**) The SMMC7721 cells and HUH7 cells were transfected with AIM2 plasmid or empty plasmid, the proliferation status of the transfected cells were detected at 0 h, 12 h, 24 h, 36 h, 48 h and 72 h by CCK8 assay. ***P < 0.001 for AIM2 transfected group compared with mock and blank control groups. (**B**) After transfection with AIM2 plasmid or empty vector, the SMMC7721 cells and HUH7 cells were plated into normal 6-well plate at the density of 1,000 cells/well and incubated for 7 days to detect the colony formation of these transfected cells.*P < 0.05. (**C**) The invasion capabilities of these AIM2 transfected cells were detected by transwell assay. **P < 0.01. (**D–F**) The HCC cells were transfected with si-AIM2 or si-NC, and proliferation of these transfected cells were detected at the indicated time points. ***P < 0.001 for si-AIM2 transfected cells compared with si-NC transfected cells (D). The colony formation capability of si-AIM2 transfected HCC cells were detected at day 7 after the transfection. *P< 0.05 for si-AIM2 transfected group compared with si-NC group (E). Transwell assay was used to determine the invasive capability of the si-AIM2 transfected cells. *P < 0.05, **P < 0.01 for si-AIM2 transfected group compared with si-NC group (F).

Our data showed that after exogenous overexpression of AIM2, SMMC7721 and HUH7 cells had significantly decreased capabilities of proliferation, colony formation and invasion compared with mock group (Figure 2A–2C); while after knockdown of AIM2 expression, the proliferation, colony formation and invasion capabilities of HepG2 and BEL7402 were significantly enhanced (Figure 2D–2F). Thus these data indicated that AIM2 could significantly suppress the malignant behaviors of HCC cells, and loss of AIM2 expression in HCC cells may contribute to HCC progression.

### AIM2 exerted its effect by forming an inflammasome and inducing pyroptosis

**Figure 3:**
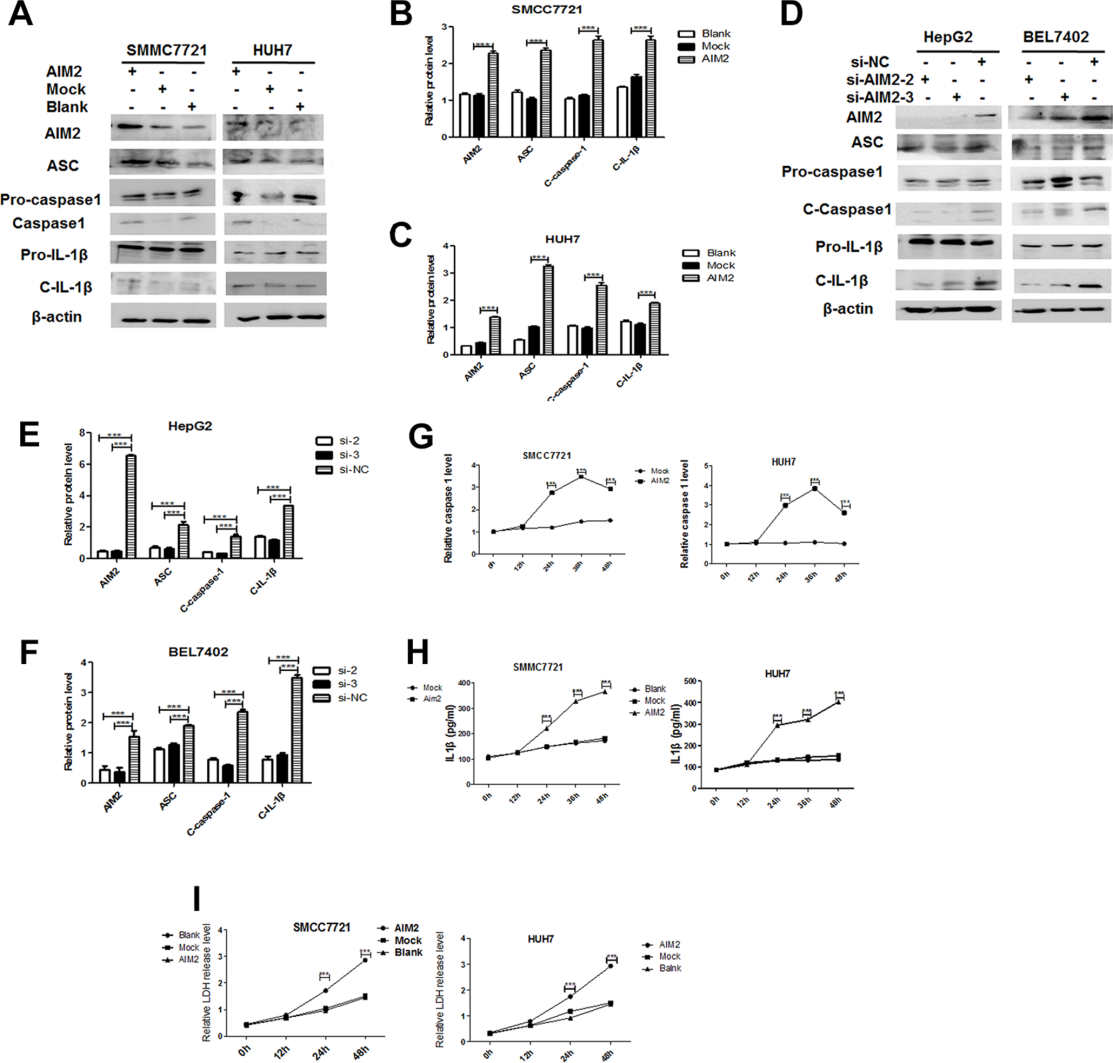
AIM2 exerted its effect by forming inflammasomes and inducing pyroptosis (**A–F**) Western blot was performed to detect the protein level of ASC, caspase-1 and IL-1β in HCC cells at 24 h after transfection with AIM2 expression plasmid (A) or interference RNA (D). Band intensities of the key proteins were analyzed in these AIM2 plasmid (B-C) or interference RNA (E-F) transfected cells (***P < 0.001, **P < 0.01, *P < 0.05 for the statistical difference for the indicated groups). (**G**) After transfection with AIM2 plasmid, Caspase-1 activity was detected by Caspase-1 Colorimetric Assay Kit at the indicated time points (left panel for SMMC7721 cells and right panel for HUH7 cells). (**H**) After transfection with AIM2 expression plasmid, the secreted IL-1β level in the supernatant was detected by ELISA. (**I**) After transfection with AIM2 plasmid, the quantity of LDH release was detected at 0 h, 24 h and 48 h after the transfection. ***P < 0.001 for AIM2 transfected group compared with mock control group.

AIM2 is known to induce the formation and activation of AIM2 inflammasome. Since ligand requirements for AIM2 inflammasome are quite permissive [12, 13], we are interested to define whether the anti-tumor effect of AIM2 in HCC cells is mediated by AIM2 inflammasome. Our data showed that after overexpression of AIM2 in HCC cells, both of the caspase-1 activation and IL-1β cleavage were significantly upregulated (Figure 3A–3C); while knockdown of AIM2 in HCC cells dramatically suppressed the cleavage of caspase-1 and IL-1β (Figure 3D–3F), which indicated that AIM2 inflammasome was formed and activated in these AIM2 overexpressed cells. Further analysis showed that overexpression of AIM2 induced a time-dependent activation of caspase-1 and IL-1β as detected by Caspase-1 activation assay (Figure 3G) and IL-1β ELISA (Figure 3H). Thus it indicated that AIM2 exerted its anti-tumor effect through AIM2 inflammasome formation.

**Figure 4:**
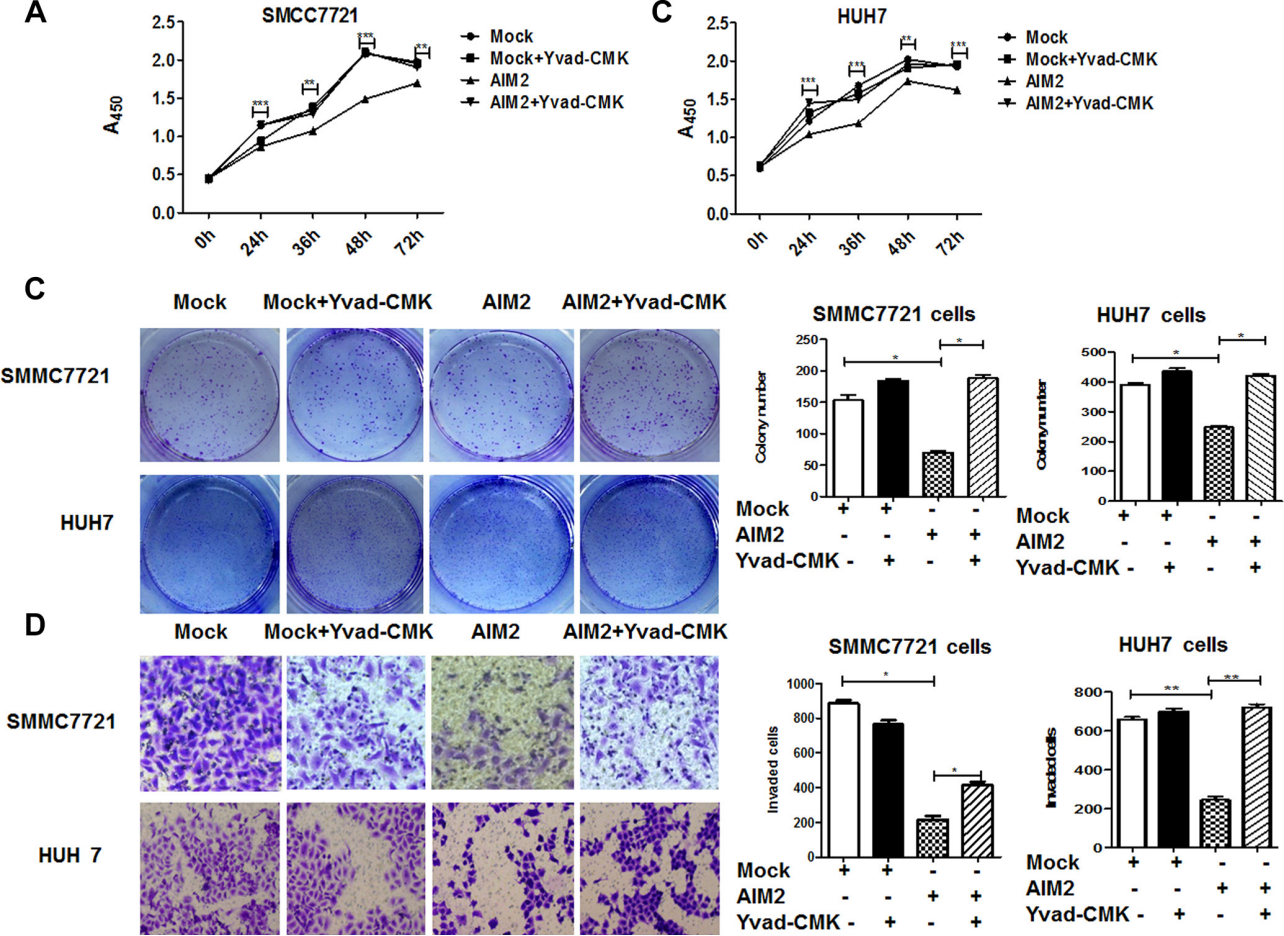
AIM2 inhibited malignant behaviors of HCC cells through forming an inflammasome SMCC7721 cells and HUH7 cells were transfected with AIM2 expression plasmid. 6 h after the transfection, inflammasome inhibitor Yvad-CMK 50 μM was put in the AIM2 transfected HCC cells. The cells transfected with empty vector were used as mock control. (**A–B**) CCK8 assay was performed to detect the proliferation of HCC cells at 0 h, 12 h, 24 h, 36 h, 48 h and 72 h after the transfection with AIM2 (A for SMMC7721 cells, B for HUH7 cells). *P < 0.05, and **P < 0.01 for statistical analysis of the proliferation of AIM2 transfected cells with and without Yvad-CMK treatment. (**C**) Colony formation capability of the AIM2 transfected cells with or without Yvad-CMK treatment was detected at day 7 after the AIM2 transfected HCC cells were plated. *P < 0.05 for comparison among indicated groups. (**D**) Transwell assay was used to determine the invasive capability of the AIM2 transfected HCC cells with or without Yvad-CMK treatment. *P < 0.05, **P < 0.01 for comparison among the indicated groups.

It was recognized that inflammasome formation and activation induced a type of programmed inflammatory cell death designated as pyroptosis. Induction of pyroptosis is characterized by activation of caspase-1, loss of integrity of cell membrane, and release of lactate dehydrogenase (LDH) which is normally maintained within the cell cytosol. Thus the release of intracellular LDH is recognized as an effective methodology to define the occurrence of pyroptosis [14], which was performed in this study to define whether pyroptosis was induced. Our data showed that pyroptosis in these AIM2 overexpressed cells was induced in a time-dependent manner (Figure 3I). Further study showed that after blocking the effect of inflammasome formation by its downstream inflammatory caspase inhibitors, suppression of malignant behaviors, including the proliferation (Figure 4A–4B), colony formation (Figure 4C) and invasion (Figure 4D) of AIM2-overexpressed HCC cells were significantly rescued. Thus, these data indicated that AIM2 exerted its anti-tumor effect on HCC cells in an inflammasome-dependent manner.

### AIM2 inflammasome suppressed mTOR-S6K1 pathway

**Figure 5:**
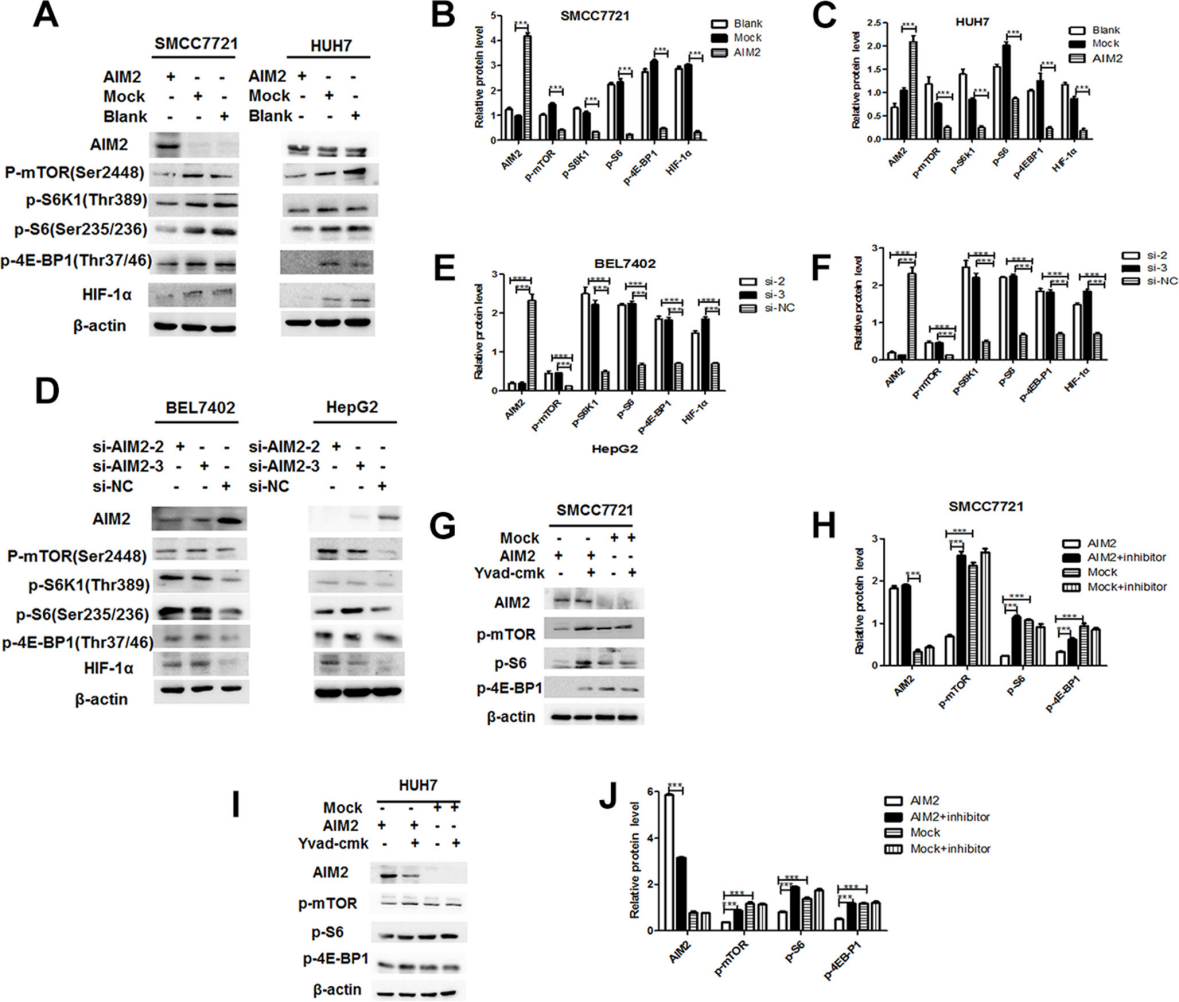
AIM2 inflammasome induced suppression of mTOR-S6K1 pathway (**A–C**) SMMC 7721 cells and HUH7 cells were transfected with EGFP-AIM2 plasmid or EGFP plasmid as mock control (A). 24 h after the transfection, activation of mTOR-S6K1-HIF1α pathway was detected by western blot, and band intensities of the key proteins were quantitatively analyzed with β-actin as an internal control and blank control group for standardization (B for SMMC7721 cells, C for HUH7 cells). (**D–F**) HepG2 cells and BEL7402 cells were transfected with AIM2 siRNA-2 or siRNA-3 to block the expression of AIM2, activation of mTOR-HIF1α pathway was detected by western blot (D), band intensities of the key proteins in mTOR-S6K1-HIF1α pathway were quantitatively analyzed with β-actin as an internal control and blank control group for standardization (E for BEL7402 cells, and F for HepG2 cells). (**G–J**) SMMC7721 cells and HUH7 cells were transfected with EGFP-AIM2 plasmid or empty plasmid before treatment with inflammasome inhibitors Yvad-CMK (50 μM) for 24 h. Western blot was used to detect the activation of mTOR-S6K1-HIF1α pathway (G for SMMC7721 cells and I for HUH7 cells). Band intensities of the key proteins were analyzed to show the differences among the indicated groups (H for SMMC7721 cells and J for HUH7 cells). ***P < 0.001 for statistical analysis of the indicated groups.

We have defined that AIM2 molecule could inhibit the malignant behaviors of HCC cells by forming AIM2 inflammasome, and then we further tried to define the molecular mechanism involved in this process. Our data showed that after overexpression of AIM2 in these HCC cells, the mTOR-S6K1 pathway was significantly suppressed as defined by the phosphorylation level of the key signaling molecules in this pathway, including p-mTOR, p-S6K1, p-S6, and p-4E-BP1. Moreover, its downstream molecule hypoxia induced factor-1α (HIF- 1α) was also significantly suppressed (Figure 5A–5C). On the contrary, block of AIM2 by its specific siRNAs significantly enhanced mTOR-S6K1 pathway activation as well as its downstream HIF-1α (Figure 5D–5F). Further analysis showed that mTOR-S6K1- HIF-1α axis was activated in a time-dependent manner (Supplementary Figure 3). It is recognized that mTOR is a nutrient sensor which promotes protein synthesis and cell proliferation, thus our data indicated that AIM2 restricted proliferation of HCC cells through suppression of mTOR-S6K1 pathway. HIF-1α is a transcription factor induced by hypoxic environment, which usually exist in fast growth tumors. Hypoxia is recognized as a selection pressure to promote cancer metastasis [15–17], and overexpression of HIF-1α in cancer cells is known to promote cancer metastasis in several reports [18–20]. Thus, suppression of HIF-1α by AIM2 may contribute to the restriction and reduction of cancer invasion of these HCC cells.

To further define whether suppression of mTOR-S6K1 pathway was mediated by AIM2 inflammasome, we detected the mTOR pathway activation after blocking inflammasome activation by its downstream caspase inhibitor, Yvad-CMK. Our data showed that suppression of mTOR pathway was significantly rescued after block of inflammasome formation (Figure 5G–5J). These data indicated that AIM2 suppressed mTOR-S6K1 pathway in an inflammasome-dependent manner.

### AIM2 inhibited malignant behaviors of HCC cells through suppression of mTOR-S6K1 pathway

**Figure 6:**
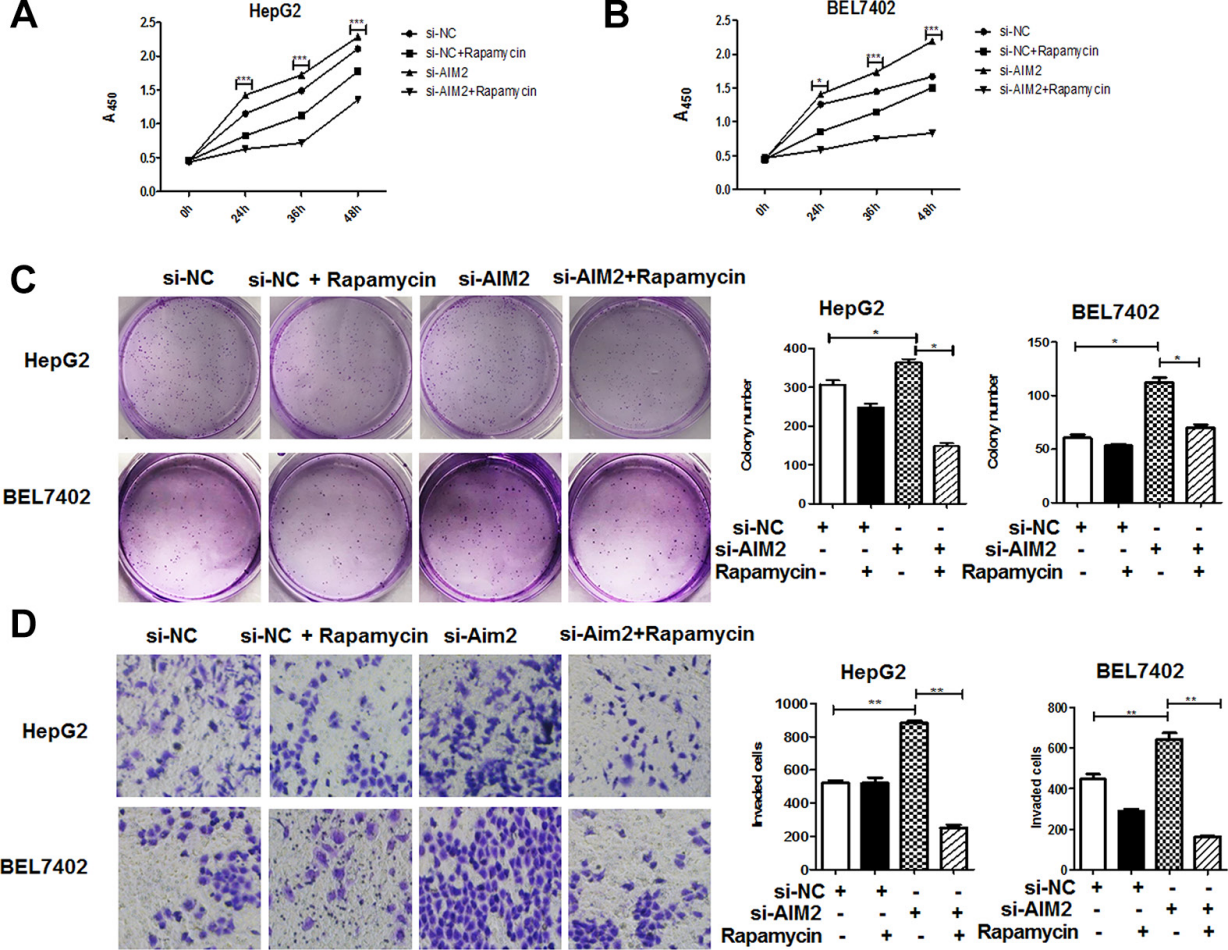
Rapamycin significantly reversed the effect of AIM2-deficiency on HCC cells HepG2 cells and BEL7402 cells were transfected with siRNA targeting AIM2 before treatment with mTOR-S6K1 pathway inhibitor rapamycin (100 nM) for 24 h. (**A–B**) CCK8 assay was performed to detect the proliferation status of the treated cells at 0 h, 24 h, 36 h, 48 h and 72 h (A for HepG2 cells and B for BEL7402 cells). ***P < 0.001 for comparison of si-AIM2 transfected cells with and without rapamycin treatment. (**C**) Colony formation assay was performed to detect the effect of rapamycin on the si-AIM2 transfected HCC cells. (**D**) Transwell assay was used to determine the effect of rapamycin on the invasive capability of si-AIM2 transfected cells. *P < 0.05, **P < 0.01 for comparison between indicated groups.

To further define whether lack of AIM2 expression in HCC cells contributed to HCC progression through loss of suppression of mTOR-S6K1 pathway, we investigated the effect of mTOR inhibitor, rapamycin on the malignant behaviors of AIM2-knocked down HCC cells. Our data showed that after treatment with rapamycin, the malignant behaviors of these AIM2 deficient HCC cells, including proliferation (Figure 6A–6B), colony formation (Figure 6C) and invasion capabilities (Figure 6D) were significantly suppressed. These data indicated that loss of AIM2 in these HCC cells promoted cancer progression through activation of mTOR-S6K1 pathway.

### Exogenous overexpression of AIM2 suppressed the tumorigenecity in nude mice

**Figure 7:**
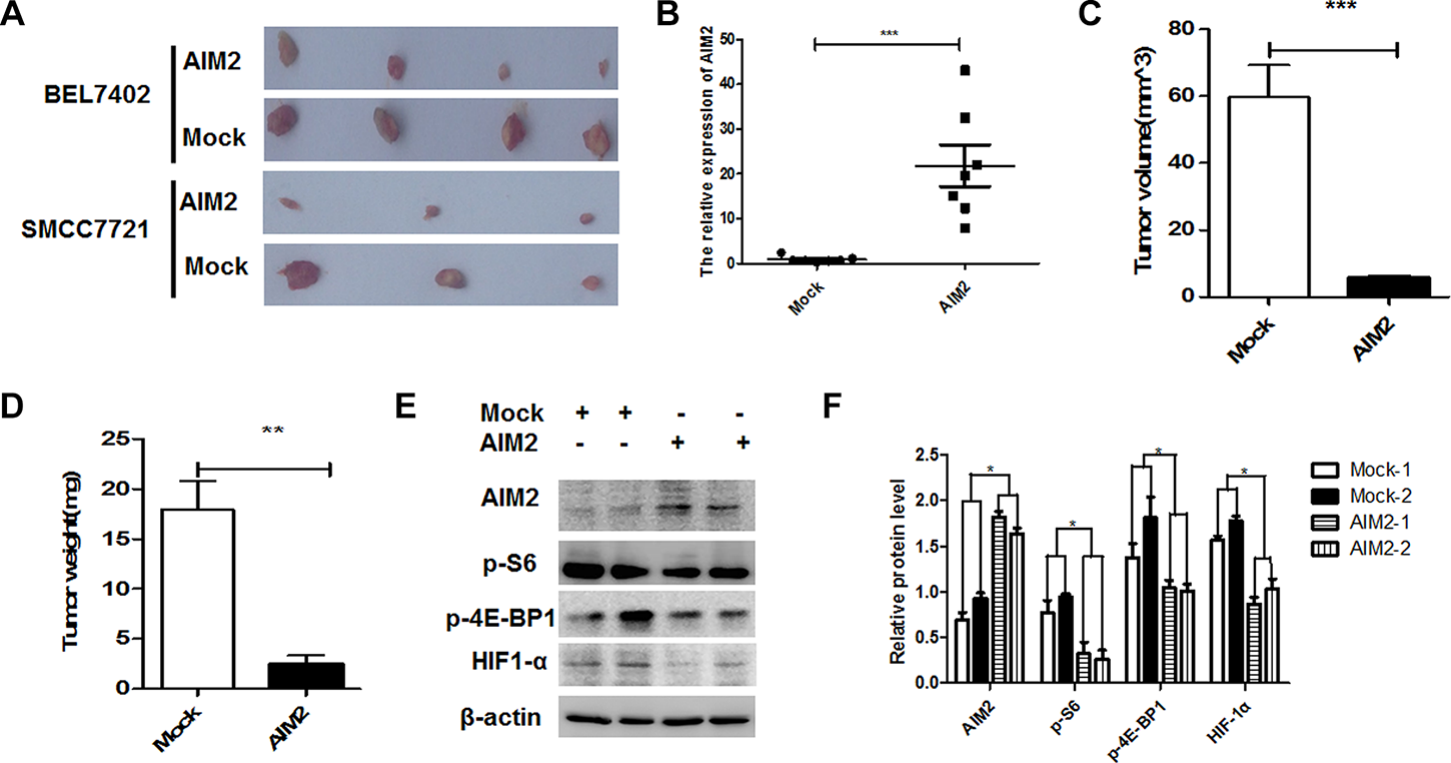
Exogenous overexpression of AIM2 suppressed xenotransplant tumor growth in nude mice BEL7402 cells and SMMC7721 cells (1 × 10^7^) were subcutaneously injected into the left upper flank of the nude mice. When visible tumor appeared, tumors were injected with 30 ug of AIM2 plasmid or empty vector. (**A**) The photo of the excised tumors from sacrificed nude mice of AIM2 plasmid transfected group and mock control group. (**B**) qRT-PCR was performed to detect and compare the AIM2 mRNA in the AIM2 overexpressed group and mock group. ***P < 0.001. (**C–D**) Statistical analysis of the tumor volume (C) and tumor weight (D) was performed between the AIM2 transfected group and mock group. (**E–F**) Western blot was performed to detect the activation of mTOR-S6K1 pathway in the AIM2 transfected group and mock group (E). Band intensities were analyzed for the presented bands and statistical differences were shown among indicated groups (F). *P < 0.05, **P < 0.01, and ***P < 0.001 for statistical differences in the indicated groups.

Our data showed that AIM2 was significantly downregulated in HCC cells, and loss of AIM2 in HCC cells promoted HCC progression through activation of mTOR pathway. To further assess the anti-tumor effect of AIM2 *in vivo*, xenograft tumor models were constructed by subcutaneous injection of BEL7402 and SMMC7721 HCC cells in nude mice. Visible tumor appeared on day 5 after the transplantation. The formed tumors were then transfected with AIM2 expression plasmid or the empty vector control every other day before the mice were sacrificed on day 25 after the transplantation. The excised tumors from each group were presented in Figure 7A. Real-time PCR verified that AIM2 was successfully overexpressed in the AIM2 plasmid transfected cells (Figure 7B). Our data showed that both of the average size and weight of the AIM2 transfected tumors were significantly decreased compared with the mock control group (Figure 7C–7D). Western blot assay showed that mTOR-S6K1 pathway was significantly suppressed in the AIM2 transfected tumors (Figure 7E–7F). These *in vivo* data further verified that AIM2 inhibited HCC growth through suppression of mTOR-S6K1 pathway.

## DISCUSSION

AIM2 is a cytosolic double-stranded DNA receptor that contributes to the host defense against pathogens by forming a multi-protein molecular platform designated inflammasome. It is recently recognized that AIM2 plays an important dual role in both innate immunity and tumor pathology, though its role in cancer is not fully clarified. Here we investigated the role of AIM2 in the progression of hepatocarcinoma and its potential involved molecular mechanism, and our data indicated a previously unrecognized role of AIM2 in HCC development and progression.

In consistence with this study, AIM2 was described to limit proliferation and tumorigenecity in colon [11, 21], breast [9], and prostate [10] cancers. It was also reported that AIM2 had a high frequency of frameshift mutations in gastric, endometrial and colorectal cancers [5]. Though the tumor suppressor role of AIM2 has been established, its function and mechanism in the progression of HCC is defined here for the first time.

In this study, our data implicated a previously unrecognized role of AIM2 in preventing HCC progression by suppressing mTOR-S6K1 pathway in dependence of inflammasome formation. mTOR, as a central sensor for cell nutrient and growth, provides a pivotal link between environmental risk factors and the cellular damage that initiates the inflammatory and regenerative responses leading to liver cancer [22–24]. Aberrant activation of mTOR-S6K1 signaling results in several aspects of dysregulation which may lead to liver cancer. First, mTOR-S6K1 is at the crossroad of metabolic and mitogenic pathways, deregulations of these pathways may result in disordered cellular proliferation and aberrant metabolism, which might predispose to malignant transformation. Second, overactivation of mTOR-S6K1 pathway causes HIF-1α activation, which may lead to the migration and invasion of cancer cells. Our data thus demonstrated that AIM2 played as a tumor suppressor in HCC cell through forming AIM2 inflammasome and further suppressing mTOR-S6K1 pathway. Absence of its expression leads to over-activation of mTOR-S6K1 pathway, which consequently leads to cancer. Though the link between mTOR-S6K1 activation and hepatocarcinogenesis has been established, suppression of mTOR-S6K1 pathway by AIM2 inflammasome is reported in this study for the first time.

Besides these experimental data, clinical investigation also supported the tumor suppressor role of AIM2 in HCC patients. We showed that lack of AIM2 expression in HCC tissues was significantly correlated with larger tumor volumes, lower differentiation status, more advanced disease stages and more metastasis, which indicated that loss of AIM2 expression in HCC patients contributed to disease progression.

Thus, our integrated data from clinical investigation, cellular model and animal model altogether demonstrated the tumor suppressor role of AIM2 in HCC. It indicated that lack of AIM2 expression endowed tumor cells proliferative and invasive capabilities, which further render cancer cells more aggressive and prone to metastasis. Taken together, these findings suggest that AIM2 is a common danger sensor in different cell types, which helps the host to be exempt from infection and transformation, and thus maintains the intracellular homeostasis and sanctity.

In summary, this study provides an integrate study including cellular model, animal model and 113 clinical HCC patients, which provides clues to understand the pathogenesis of hepatocarcinoma as well as new strategies for manipulation of liver cancer. Our results suggested that lack of AIM2 expression in HCC cells contributed to disease progression of cancer. Further *in vivo* and *in vitro* studies demonstrated that exogenous overexpression of AIM2 inhibited malignancies of HCC cells through suppressing mTOR-S6K1 pathway, which suggested a protective role of AIM2 against liver cancers. Therefore, it indicated that therapeutic strategy by upregulating AIM2 may pave a new avenue for manipulating liver cancer, and pharmacological targeting of mTOR-S6K1 pathway may be beneficial in AIM2-deficient tumors.

## MATERIALS AND METHODS

### Clinical specimens

Paired samples of HCC tissues and corresponding non-cancerous liver tissues from 113 HCC patients taken from surgical excision specimen in the Department of Hepatobiliary Surgery of the Provincial Hospital Affiliated to Shandong University were included in this study. Among these, 49 pairs of matched tissue samples were used for immunohistochemistry (IHC) assay, and 64 pairs of matched cancer and non-cancerous tissue were used for western blot and quantitative real-time PCR assay (qRT-PCR). All the protocols dealing with the patients conformed to the ethical guidelines of the Helsinki Declaration and were approved by Shandong University Research Ethics Committee. Written informed consent was obtained from each patient before participation and approved by ethics committee of Shandong University. Details of the clinicopathologic characteristics of these recruited HCC patients were shown in Table 3.

**Table 3:** Clinicopathological characteristics of the investigated HCC patients

	Cohort 1 (*n* = 49)	Cohort 2 (*n* = 64)
Characteristics	No. of patients (%)	No. of patients (%)
Gender		
Male	44 (85.7%)	54 (83.7%)
Female	5 (14.3%)	10 (16.3%)
Age		
< 54	22 (44.9%)	30 (46.9%)
≥ 54	27 (55.1%)	34 (53.1%)
Tumor size		
< 5 cm	18 (36.7%)	33 (51.5%)
≥ 5 cm	31 (63.3%)	31 (48.5%)
Liver cirrhosis history		
Yes	42 (85.7%)	51 (79.6%)
No	7 (14.3%)	13 (21.4%)
TNM stage		
I	11 (22.4%)	25 (39.1%)
II	23 (46.9%)	14 (14.1%)
III	15 (30.7%)	21 (32.8%)
IV	0	9 (13.2%)
Regional lymph nodes		
N0	35 (71.4%)	25 (39.1%)
N1	14 (28.6%)	39 (60.9%)
BCLC stage		
0	4 (8.2%)	14 (21.8%)
A	20 (40.8%)	19 (29.7%)
B	10 (20.4%)	8 (12.5%)
C	14 (28.6%)	23 (36.0%)
D	1 (2.0%)	0
Distant metastasis		
No	28 (78.4%)	20 (21.2%)
Yes	9 (21.6%)	44 (68.8%)

### Western blot

Western blot assay to detect the protein level of AIM2 and its related signaling pathways was done as described previously [25]. Specific primary antibodies against AIM2 (#12948), p-S6K1 (#9208), p-S6 (#4858), p-4E-BP1 (#2855), HIF-1α (#3716), and caspase-1 (#2225) were bought from Cell Signaling Co.(Cell Signaling Technology, Beverly, USA). The antibodies against IL-1β(60136-1-Ig) and ASC(10500-1-AP) were bought from Proteintech (Proteintech, Chicago, USA). Expression of β-actin was analyzed as a loading control to compare the relative expression of indicated proteins.

### RNA extraction and quantitative real-time PCR

RNA was extracted from the specimens by TRIzol (Invitrogen, Massachusetts, USA). 2 ug of RNA was transcribed into cDNA using FastQuant RT Kit (TIANGEN, Beijing, China) according to the manufacture's protocol. Expressions of objective genes were detected by qRT-PCR using QuantiFast SYBR Green PCR Kit (Qiagen, MD, USA) following the manufacturer's instructions. Primers for the AIM2 gene were forward 5′- ATCAGGAGGCTGATCCCAAA -3′, reverse 5′- TCT GTTCAGGCTTAACATGAG -3′.

Reactions of qRT-PCR were performed using LightCycler CFX96 (Bio-Rad, CA, USA) according to the manufacturer's instructions. A melting-curve analysis was performed to ensure specificity of the products. β-actin was analyzed as an internal control. The relative mRNA levels of target genes were obtained by using the 2 ^−ΔΔCt^ method with all assays performed in triplicate.

### Immunohistochemical staining and evaluation

Immunohistochemical analysis was carried out to assess the location and expression of AIM2 in liver cancer tissues and corresponding non-cancerous liver tissues. Paraffin sections were baked at 65°C for 2 hours followed by hydration in graded alcohol as previously described [25]. The slides were incubated with AIM2 monoclonal antibody (#ab93015, Abcam, Cambridge, MA, USA) for incubation at 4°C overnight, and then performed according the procedure described in the SP9000 kit and 3,3′-diaminobenzidine (DAB) kit (ZSGB-BIO, Beijing, China). High resolution pictures were obtained by Olympus digital electron microscope and DP2-BSW controller software (Olympus, Tokyo, Japan). Immunoreactivities of the immunohistochemical staining were evaluated as described before [25].

### Cell culture and transfection

Human HCC cell lines including MHCC97H, MHCC97L, BEL7402, SMCC7721, HepG2 and HUH7 were used in this study. BEL7402 and SMCC7721 cells were cultured in RPMI1640 medium (Hyclone, Massachusetts, USA) supplemented with 10% Fetal Bovine Serum. HepG2 and HUH7 cells were cultured in DMEM-High Glucose Medium (Hyclone, Massachusetts, USA) supplemented with 10% Fetal Bovine Serum. pEGFP-N1-hAIM2 plasmid was from Dr. Emad Alnemri at Thomas Jefferson University. The small interfering RNA targeting hAIM2 was synthesized by RIBOBIO (RIBOBIO, Guangzhou, China).

### Cell proliferation, colony formation, and invasion assay

Cell proliferation activities were measured by Cell Counting Kit 8 (CCK8, Dojindo, Tokyo, Japan) according to its protocol. The proliferation status of cells were detected at different time points, including 0, 12 h, 24 h, 36 h, 48 h, and 72 h. Cell invasion and colony formation assay were performed as described before [26, 27].

### ELISA, Caspase-1 activation assay, and LDH release assay

Standard sandwich ELISA were used to detect the levels of IL-1β in the culture supernatant of human HCC cell lines by Human IL-1β pre-coated ELISA kit (DAKEWE, Beijing, China) according to the manufacture's direction. Cell lysates were used to test caspase-1 activity by Caspase-1 Colorimetric Assay Kit (Biovision, California, USA) according to the manufacture's protocol. LDH release assay was performed by the LDH release assay kit (Beyotime Biotechnology, Beijing, China) according to the manufacture's instruction.

### *In vivo* tumor growth assay

5-week old immunodeficient male BALB/c athymic nude mice were purchased from Huafukang Biotechnology Ltd (Huafukang, Beijing, China) and housed in an aseptic environment in Shandong University Experimental Animal Centre. The mice were randomly divided into two groups for BEL7402 cells and SMMC7721 cells injection, respectively. HCC cells (1 × 10^7^) were suspended in 100 ul serum-free RPMI1640 medium and subcutaneously injected to the left upper flank of each mouse. Formed tumors were injected with 30 ug of EGFP-AIM2 plasmid or empty vector to the tumor once every 3 days before the mice were sacrificed by cervical dislocation. Weight and volume of the excised tumor were analyzed immediately after the mice were sacrificed. The tumor volume was determined using a caliper and calculated by the formula: V (mm^3^) = Width^2^ (mm^2^) × Length (mm)/2. All animal protocols were reviewed and approved by Animal Ethics Committee of Shandong University, and Laboratory Animal Management Committee of Shandong University.

### Statistical analysis

*Χ*^2^-test and *t*-test were applied to compare categorical variables and continuous variables, respectively. Pearson correlation assay was used to evaluate continuous variables and Spearman's rank correlation test was used to evaluate correlations between ranked variables. Data were analyzed using SPSS 16.0 (SPSS, IL, USA) and a *P*-value of 0.05 was considered statistically significant.

## SUPPLEMENTARY MATERIALS FIGURES


