# Supplementary material for: Loss of AIM2 expression promotes hepatocarcinoma progression through activation of mTOR-S6K1 pathway
Source: Oncotarget. 2016 May 4;7(24):36185–97. doi: 10.18632/oncotarget.9154 (PMC5094992; doi:10.18632/oncotarget.9154)
Supplement: Supplementary file 1 [file oncotarget-07-36185-s001.pdf]

# Loss of AIM2 expression promotes hepatocarcinoma progression through activation of mTOR-S6K1 pathway

## Supplementary Materials

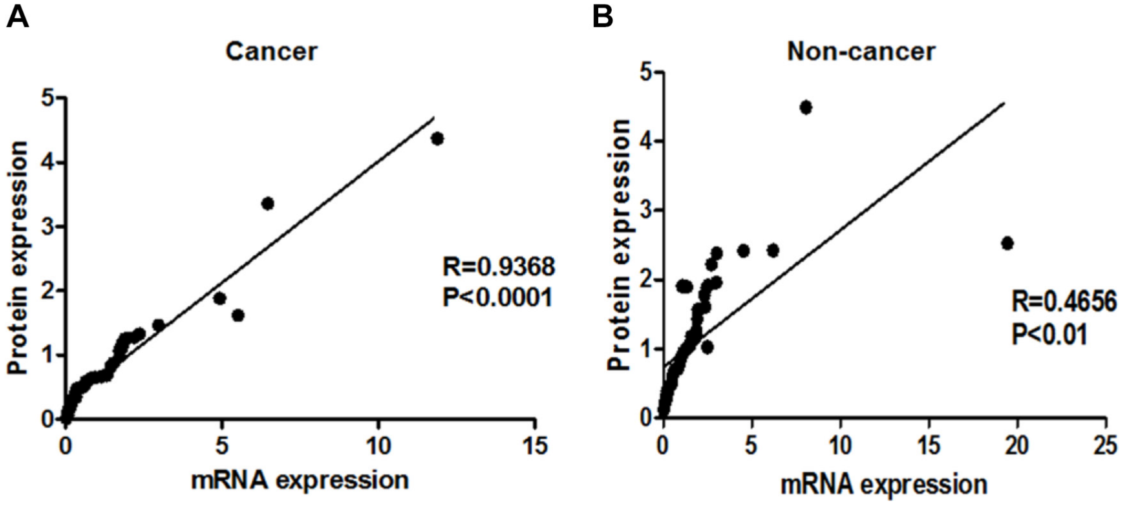


**Supplementary Figure S1: Correlation analysis of the mRNA and protein level of AIM2 expression.** (**A–B**) AIM2 mRNA and protein expression were detected by real-time PCR and western blot, respectively. Correlation of AIM2 mRNA and protein levels in liver cancer tissues (A) and non-cancerous liver tissues (B) were presented.

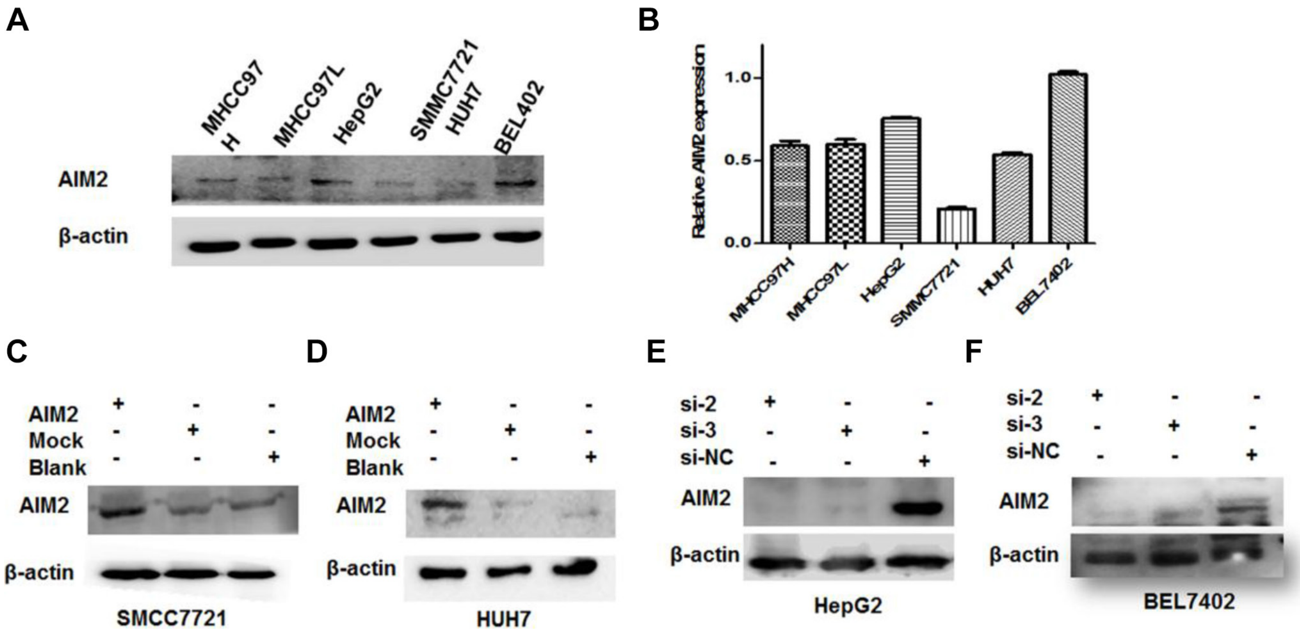


**Supplementary Figure S2: AIM2 expression was detected in six human liver cancer cell lines.** (**A**) Western blot was used to determine the AIM2 protein expression in 6 human HCC cell lines. (**B**) Band intensity analysis was done to compare the relative level of AIM2 in these 6 detected HCC cell lines. (**C–D**) SMCC7721(C) and Huh7 cells (D) were transfected with 2ug EGFP-AIM2 plasmid to confirmed the efficiently overexpression of AIM2 in these HCC cells. Cells transfected with empty vectors were used as mock control. (**E–F**) Western blot was performed in two AIM2 highly expressed HCC cell lines(E for HepG2 cells and F for BEL7402 cells ) to detect the inhibitory efficacy of three synthesized siRNA(siRNA-1and siRNA-3) specifically targeting AIM2.

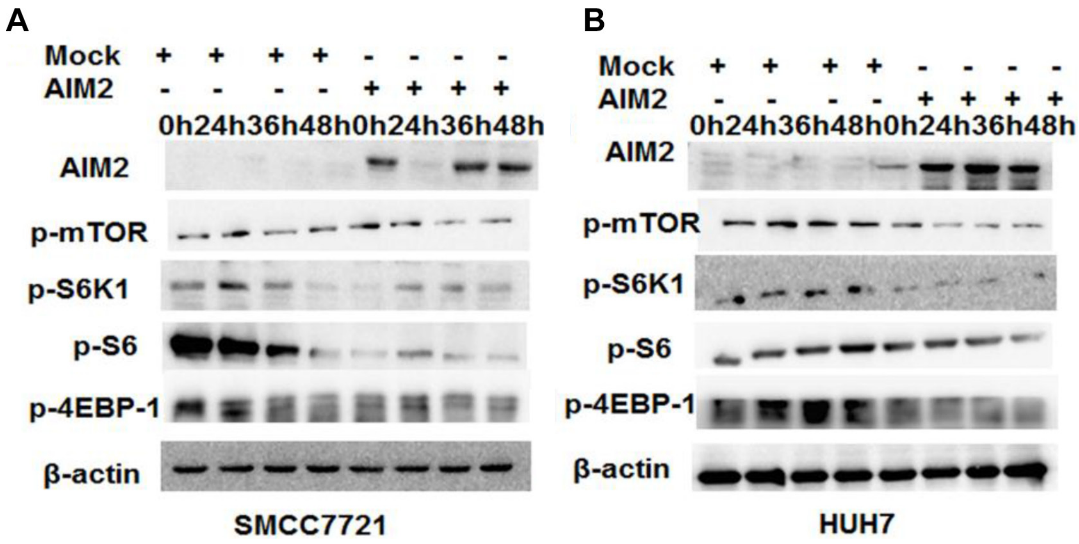


**Supplementary Figure S3: Effect of exogenous overexpression of AIM2 on the activation of mTOR-S6K1 pathway.** (**A–B**) After transfection of AIM2 expression plasmid or mock plasmid to SMCC7721(A) and HUH7 (B) cells, the cells were further cultured for 0 h, 24 h, 36 h and 48 h before harvested for western blot analysis of the mTOR-S6K1 pathway activation.
